# Factor H facilitates the clearance of GBM bound iC3b by controlling C3 activation in fluid phase

**DOI:** 10.1016/j.molimm.2009.03.030

**Published:** 2009-06

**Authors:** Danielle Paixão-Cavalcante, Steven Hanson, Marina Botto, H. Terence Cook, Matthew C. Pickering

**Affiliations:** a Molecular Genetics and Rheumatology Section, Faculty of Medicine, Imperial College, Hammersmith Campus, Du Cane Road, London, W12 0NN, UK; b Department of Histopathology, Faculty of Medicine, Imperial College, Hammersmith Campus, Du Cane Road, London, W12 0NN, UK

**Keywords:** Dense deposit disease, Factor H-deficient mice, Factor H reconstitution, Restore C3 regulation in plasma

## Abstract

Dense deposit disease (DDD) is strongly associated with the uncontrolled activation of the complement alternative pathway. Factor H (CFH)-deficient (C*fh*^−/−^) mice spontaneously develop C3 deposition along the glomerular basement membrane (GBM) with subsequent development of glomerulonephritis with features of DDD, a lesion dependent on C3 activation. In order to understand the role of CFH in preventing renal damage associated with the dysregulation of the alternative pathway we administered purified mouse CFH (mCFH) to C*fh*^−/−^ mice. 24 h following the administration of mCFH we observed an increase in plasma C3 levels with presence of intact C3 in circulation showing that mCFH restored control of C3 activation in fluid phase. mCFH resulted in the reduction of iC3b deposition along the GBM. The exogenous mCFH was readily detectable in plasma but critically not in association with C3 along the GBM. Thus, the reduction in GBM C3 was dependent on the ability of mCFH to regulate C3 activation in plasma. Western blot analysis of glomeruli from C*fh*^−/−^ mice demonstrated the presence of iC3b. Our data show that the C3 along the GBM in C*fh*^−/−^ mice is the C3 fragment iC3b and that this is derived from plasma C3 activation. The implication is that successful therapy of DDD is likely to be achieved by therapies that inhibit C3 turnover in plasma.

## 1 Introduction

DDD is an inflammatory renal disease that is strongly associated with complement dysregulation. Around 50% of individuals with DDD will reach end-stage renal failure 10 years after the onset of the disease (Smith et al., 2007). DDD is characterized by electron-dense transformation of the glomerular basement membrane (GBM). The precise composition of the electron-dense areas is unknown but the margins of the deposits contain complement components C3, C5 and C9 in the absence of immunoglobulin (Sethi et al., 2009). DDD is strongly associated with dysregulation of the alternative pathway (AP) of complement activation and specifically with impairment of the function of the plasma protein complement factor H (CFH), the major physiological regulator of the AP. Moreover, DDD can also be triggered by the presence of C3 nephritic factor, an autoantibody that stabilizes the AP C3 convertase (an enzyme complex that cleaves C3), anti-factor H antibodies and dysfunctional C3 molecules (Linshaw et al., 1987; Marder et al., 1983; Meri et al., 1992). The enhanced AP activation in all these situations results in depletion of C3 in plasma.

Complement component C3 is a complex glycoprotein consisting of *α* and *β* chains containing a thioester bond responsible for its covalent binding to hydroxyl or amine groups present on target surfaces (Dodds et al., 1996; Sahu et al., 1994). C3 can assume four different conformational states governed by thioester bond rearrangement and release of small proteolytic fragments (Nishida et al., 2006). The generation of C3b, via proteolytic cleavage by C3 convertase, initiates a positive feed-back loop that amplifies the activation of the complement cascade. However, this system requires a strict regulatory mechanism to prevent host cell injury and to maintain the normal physiological functions of the complement system. Factor I (CFI) in the presence of its cofactors (CFH, MCP [membrane cofactor protein, CD46] or CR1 [complement receptor one, CD35]) inactivates C3b via cleavage of the C3 *α*-chain, releasing a 17-amino acid peptide termed C3f, forming iC3b. CFI also further degrades iC3b into C3c and C3dg using CR1 as a cofactor (Harrison and Lachmann, 1980).

Previously, we generated homozygous CFH-deficient mice (C*fh*^−/−^) enabling the contribution of CFH in the development of DDD to be investigated in an *in vivo* experimental model. C*fh*^−/−^ mice developed uncontrolled AP activation with reduced concentration of plasma C3 and presence of C3 breakdown products in circulation. In addition, they developed C3 deposition along the GBM and a glomerular lesion morphologically similar to human DDD (Pickering et al., 2002). Intercrossing C*fh*^−/−^ mice with mice deficient in factor B prevented the development of DDD demonstrating that the spontaneous renal disease was dependent on C3 activation through the AP (Pickering et al., 2002). Intercrossing C*fh*^−/−^ mice with mice deficient in CFI (*Cfi*^−/−^) also prevented the development of DDD demonstrating that the spontaneous renal disease was dependent on the ability of factor I to cleave C3b to iC3b (Rose et al., 2008).

Current therapeutic approaches to DDD are aimed at slowing the progression of the renal damage by decreasing proteinuria, improving renal hemodynamics and limiting leukocyte infiltration in the kidney (Smith et al., 2007). DDD due to the generation and persistence of autoreactive antibodies (C3Nef and anti-CFH antibodies) would theoretically be amenable to treatments that inhibit the differentiation, maturation, and allostimulatory function of B and T lymphocytes. For individuals with CFH mutations, CFH replacement therapy would seem the most logical approach. In this respect it is notable that CFH-deficient patients with atypical haemolytic uremic syndrome have shown improvement in renal function following infusions of plasma (Nathanson et al., 2001).

Here, we investigated the effects of exogenous murine CFH (mCFH) on plasma and renal C3 regulation in C*fh*^−/−^ mice. Administration of mCFH restored plasma C3 regulation in C*fh*^−/−^ mice. It also altered the renal C3 staining pattern. Exogenous mCFH resulted in reduction in GBM C3 staining together with the appearance of mesangial and tubulo-interstitial C3 staining. The exogenous mCFH was readily detectable in plasma but could not be detected in association with C3 along the GBM. Thus, the reduction in GBM C3 was dependent on the ability to regulate C3 activation in plasma. Moreover, in this study we provide further evidence that iC3b is the fragment of C3 present along the GBM in C*fh*^−/−^ mice.

## 2 Material and methods

### 2.1 Animals

C*fh*^−/−^ mice were generated previously (Pickering et al., 2002). *CD11b*^−/−^ mice were purchased from the Jackson laboratory. Mice deficient for both CFH and CD11b (C*fh*^−/−^*CD11b*^−/−^) were generated by inter-crossing C*fh*^−/−^ and *CD11b*^−/−^ mice. All experimental animals were age and sex-matched and were bred on to the C57BL/6 genetic background for at least 10 generations. All experimental procedures were done in accordance with institutional guidelines.

### 2.2 Purified mouse CFH (mCFH)

mCFH was purified from purchased whole serum (Innovative Research, MI, USA) using heparin affinity chromatography. Briefly, serum was treated with 7% polyethylene glycol 8000 (Sigma, Gillingham, UK) on ice. The pellet was dissolved in PBS, dialyzed against Tris–NaCl (Tris 20 mM; NaCl 50 mM pH 7.4) and applied to heparin affinity chromatography (Heparin Sepharose™ 6 Fast Flow, Amersham Pharmacia Biotech, Uppsala, Sweden). After extensive washing, proteins were eluted with a linear salt gradient (75–250 mM NaCl). CFH-containing fractions were pooled, dialyzed against Tris–HCl (20 mM, pH 8.6) and subjected to ion exchange chromatography (DEAE-sepharose™ Fast Flow, GE Healthcare Bio-Sciences AB, Uppsala, Sweden). The column was extensively washed and the bound proteins eluted with a linear salt gradient (0–300 mM NaCl). CFH-containing samples were pooled and dialyzed against PBS. After each chromatography step the samples were checked for the presence of CFH by western blot using polyclonal cross-reactive anti-human CFH (Quidel, CA, USA). The purity of the samples was assessed by Coomassie stained SDS-PAGE gels. Our preparations contained a major protein band at the expected molecular weight position for CFH. Purified CFH samples used in these experiments contained approximately 0.4 μg/mL of lipopolysaccharide (LPS) assayed using Limulus Amebocyte Lysate (LAL) QCL-1000 (Cambrex, MD, USA).

### 2.3 Tagging mCFH with Alexa Fluor 488

Purified mCFH was tagged with a fluorescein analogue dye, Alexa 488 by the interaction of tetrafluorophenyl ester moiety with primary amines present in CFH forming stable dye-protein conjugated according to manufacturer's instructions (Invitrogen, Paisley, UK). The efficiency of the tag was measured by spectrophotometry.

### 2.4 Administration of mCFH to C*fh*^−/−^ mice

Animals were intraperitoneally (i/p) injected with 1 mg of purified mCFH preparation or an identical volume of phosphate-buffered saline (PBS). In view of the presence of LPS in the mCFH preparations groups of animals were also injected with purified LPS (*E. coli* O111:B4, Sigma–Aldrich Co., Gillingham, UK) to identify any LPS-dependent effects. Twenty-four hours after the injection mice were sacrificed and plasma and renal tissue collected.

### 2.5 Depletion of neutrophils in vivo

To achieve neutrophil depletion *in vivo* C*fh*^−/−^ mice were injected i/p at day 0 with 0.5 mg of rat monoclonal IgG_2b_ anti-murine neutrophil antibody (Ly.6G, Santa Cruz Biotechnology, CA, USA). 24 h later mice received either mCFH (1 mg), LPS (0.75 μg) or PBS. Mice were sacrificed on day 2. Blood samples collected before and 24 h after administration of Ly.6G were analysed to confirm successful neutrophil depletion using FACS and peripheral blood film examination. Peripheral blood was collected in 5% EDTA and neutrophils were stained using Phycoerythrin-conjugated rat IgG_2b_ anti-mouse GR-1 (Euro Bioscience GmbH, Friesoythe, Germany) antibody in the presence of a saturating concentration of the monoclonal antibody 2.4G2 which blocks both Fc*γ*RII and III receptor sites. The results were analyzed with a FACS Calibur instrument (Becton Dickinson, CA, USA). Peripheral blood films were prepared using a drop of peripheral blood collected in EDTA and the neutrophils identified by their characteristic nuclear morphology.

### 2.6 Plasma mCFH detection

mCFH in the peripheral blood was detected by western blot using a cross-reactive polyclonal goat antibody against human CFH (Quidel, CA, USA).

### 2.7 Plasma C3 levels

C3 levels were measured by ELISA using goat anti-mouse C3 antibody (MP Biomedicals, OH, USA) as previously described (Pickering et al., 2007). Results were quantified by reference to a standard curve generated from acute phase sera containing a known quantity of C3 (Calbiochem, CA, USA).

### 2.8 Analysis of glomerular C3

Glomeruli were isolated from frozen sections using a laser capture microscope (Leica, Wetzlar, Germany) and dissolved in 10% SDS solution containing protease inhibitor cocktail (Sigma–Aldrich Co., Gillingham, UK). Solubilised dissected glomerular tissue was subjected to SDS-PAGE under reduced condition. C3 was then detected by western blot using a polyclonal goat antibody against mouse C3 (MP Biomedicals, CA, USA).

### 2.9 Histological studies

Kidneys were fixed in Bouin's solution (Sigma, Gillingham, UK) and sections stained with periodic acid-Schiff reagent. Glomerular histological analysis and neutrophil counting were performed in a blinded manner as previously described (Pickering et al., 2006). Twenty glomeruli were analysed per section. For immunostaining kidneys were snap-frozen and sections cut and fixed in acetone. C3 was detected using either FITC-conjugated goat anti-mouse C3 (MP Biomedicals, CA, USA) or biotinylated-goat anti-mouse C3d (R&D system, MN, USA).

### 2.10 C3 expression

Total RNA was isolated from whole kidney and liver of mice injected with PBS or mCFH and reverse transcribed into cDNA. Real-time polymerase chain reaction assays were performed on an ABI 7500 Sequence Detection System (Applied Biosystems, Warrington, UK) using SYBER Green (Stratagene, Cambridge, UK). The assay was carried out in triplicate using intron-spanning primer sequences for C3 (forward primer 5′CACCGCCAAGAATCGCTAC-3′; reverse primer 5′GATCAGG TGTTTCAGCCGC-3′), 18S (forward primer 5′CCGCAGCTAGGAATAATGGAA T-3′; reverse primer 5′CGAACCTCCGACTTTCGTTCT-3′) and GAPDH (forward primer 5′CACTCTTCCACCTTCGATGC-3′; reverse primer 5′AGGGAGATGCTC AGTG TTGG-3′). The relative expression level of C3 was determined by using the 2^−ΔΔCt^ method (Arocho et al., 2006).

### 2.11 Statistical analysis

The Mann–Whitney *U* test was used for comparison of two groups, whilst for analysis of three or more groups Bonferroni's multiple comparison test was used. Data were analyzed using GraphPad Prism version 3.0 for Windows (GraphPad, San Diego, USA).

## 3 Results

### 3.1 Administration of mCFH regulates plasma C3 activation in C*fh*^−/−^ mice

**Fig. 1:**
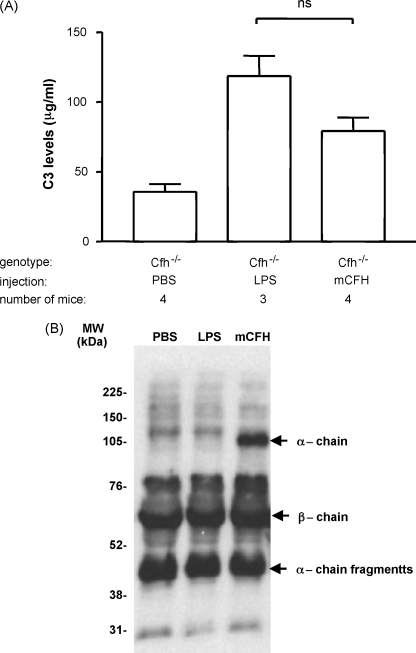
Plasma C3 levels and state in C*fh*^−/−^ mice injected with purified mCFH. (A) Plasma C3 levels in C*fh*^−/−^ mice 24 h after the injection of PBS, 1 mg of purified mouse factor H or 0.75 μg of LPS. Wild-type C3 control level in this ELISA was 387 μg/ml. Columns denote median values with standard deviation (B) Western blot for mouse C3 using EDTA plasma from C*fh*^−/−^ mice 24 h after the injection of PBS, mCFH (1 mg) or LPS (0.75 μg) under reducing conditions. The EDTA plasma dilution used for all the samples was 1 in 100. LPS: lipopolysaccharide, PBS: phosphate-buffered saline.

To investigate whether the administration of purified mCFH could restore control of AP activation in plasma we assessed the level and state of circulating C3 in C*fh*^−/−^ mice after mCFH administration. Plasma C3 levels in unmanipulated C*fh*^−/−^ mice are markedly reduced with median levels of approximately 5% of wild-type levels (Pickering et al., 2002). Administration of 1 mg of our purified mCFH to C*fh*^−/−^ mice resulted in an increase in plasma C3 levels at 24 h (Fig. 1a). Since our mCFH preparation contained LPS we also assessed plasma C3 levels in three C*fh*^−/−^ mice that received 0.75 μg (twice the amount of LPS detected in the administered mCFH preparation) of purified LPS alone. At 24 h these mice had an increase in plasma C3 levels similar to C*fh*^−/−^ mice that had received mCFH (Fig. 1a). We next assessed the activation state of the plasma C3 using western blotting under reducing conditions (Fig. 1b). This allowed identification of C3 *α*-chain fragments thereby enabling us to discriminate between intact C3 and its proteolytic fragments (C3b, iC3b and C3dg). Intact *α*-chain was only detectable in the C*fh*^−/−^ mice that had received mCFH (Fig. 1b, far right lane). In contrast, in C*fh*^−/−^ mice injected with LPS or PBS no intact C3 *α*-chain was present. In these animals, the C3 *β*-chain was present together with C3 *α*-chain fragments consistent with ongoing plasma C3 activation. Taken together, this data shows that whilst either LPS or mCFH can increase total antigenic C3 levels in plasma, only mCFH was able to regulate AP activation allowing intact plasma C3 to circulate in the C*fh*^−/−^ mice.

### 3.2 The presence of mCFH influences C3 localization in the kidney

**Fig. 2:**
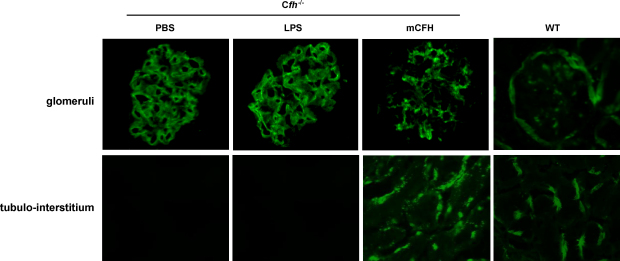
Renal C3 stain in C*fh*^−/−^ mice 24 h after the injection of purified mCFH. Kidney sections were stained for C3 using polyclonal anti-mouse C3. Linear capillary wall staining is evident in C*fh*^−/−^ mice injected with PBS or 0.75 μg of LPS. In contrast, mesangial staining is seen in C*fh*^−/−^ mice 24 h after injection of 1 mg of mCFH. C3 staining is present along Bowman's capsule and within tubulo-interstitium of wild-type mice. Tubulo-interstitial staining is absent in C*fh*^−/−^ mice injected with PBS or LPS. However, after administration of mCFH, tubulo-interstial staining is now evident in the C*fh*^−/−^ animals. Original magnification 40×. WT: wild-type.

Unmanipulated C*fh*^−/−^ mice have marked deposition of C3 along the GBM (Pickering et al., 2002). Therefore, we next examined whether administration of mCFH could influence glomerular C3 in C*fh*^−/−^ mice. 24 h following mCFH administration, we detected striking changes in the glomerular staining pattern for C3 in the C*fh*^−/−^ mice (Fig. 2). C3 was hardly detectable on the GBM using the polyclonal anti-mouse C3 antibody. In addition, C3 staining was now evident within the mesangium and within the tubulo-interstitium (Fig. 2). In contrast, the pattern of C3 staining in C*fh*^−/−^ mice injected with LPS or PBS did not differ from the unmanipulated C*fh*^−/−^ mice.

**Fig. 3:**
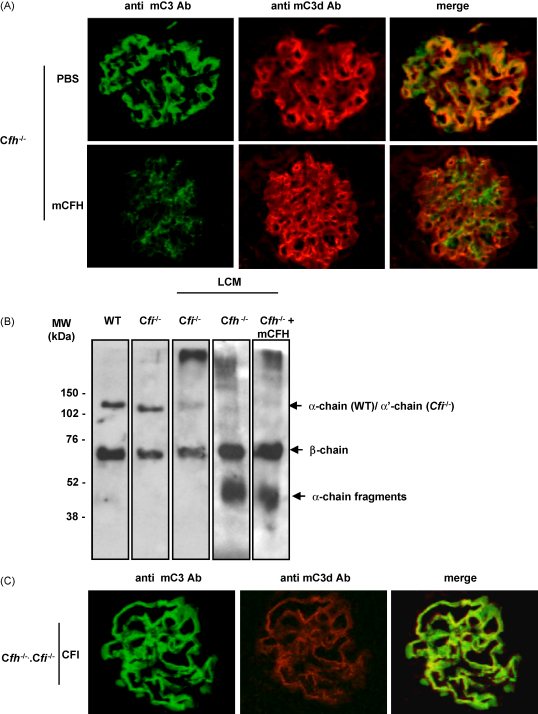
Nature of C3 bound to the glomeruli 24 h after the administration of mCFH. (A) Kidney sections were stained for C3 using polyclonal anti-mouse C3d (red stain) or polyclonal anti-mouse C3 (green stain) antibodies. In the mice injected with PBS linear capillary wall staining was evident using both antibodies. However, in the C*fh*^−/−^ mice injected with mCFH a mesangial staining pattern was evident using anti-mouse C3 antibody whilst the linear capillary wall staining pattern remained unchanged with the anti-C3d antibody. The merged images showed that the areas of mesangial reactivity did not co-stain with the anti-C3d. Original magnification 40×. (B) Western blot analysis of C3 under reducing conditions using solubilised laser dissected glomerular tissue from C*fi*^−/−^, C*fh*^−/−^ or C*fh*^−/−^ mice injected with mCFH. To demonstrate the positions of the intact C3 *α*-chain, the *α*′ chain of C3b and the *β*-chain of C3 plasma from both wild-type (intact C3) and C*fi*^−/−^ mice (in which all C3 is circulating as C3b) was also run on the gel. C3 *α*-chain fragments and the C3 *β*-chain were evident in C*fh*^−/−^ mice with or without the administration of mCFH. In contrast no *α*-chain fragments were evident in glomeruli from C*fi*^−/−^ animals. (C) Glomerular C3 staining in mice with combined deficiency of CFH and CFI (C*fh*^−/−^.C*fi*^−/−^) that have been given CFI. Following adminstration of sera containing CFI, linear capillary wall C3 staining develops that is reactive with both the anti-C3 and anti-C3d antibody. Original magnification 40×. (For interpretation of the references to color in this figure legend, the reader is referred to the web version of the article.)

In order to further define the nature of C3 that was detected in the mesangium after administration of mCFH we immunostained kidney sections using a polyclonal anti-mouse C3d antibody. Our data has shown that this antibody does not recognise intact C3 or C3b (Leung at al paper submitted). Using this antibody, GBM C3 staining was detectable to an equivalent intensity in C*fh*^−/−^ mice injected with either PBS or mCFH (Fig. 3a). However, in the C*fh*^−/−^ mice injected with mCFH, this antibody did not recognize C3 within the mesangium that was evident using the polyclonal anti-C3 antibody (Fig. 3a). This was clearly seen when the two staining images were merged. This showed complete overlap of anti-C3 and anti-C3d glomerular staining pattern in C*fh*^−/−^ mice injected with PBS. In contrast, there was no overlap of mesangial and GBM staining patterns in the sections from C*fh*^−/−^ mice injected with mCFH (Fig. 3a). This suggested that the nature of the C3 along the GBM was not the same as that of the C3 within the mesangium.

To further investigate this we isolated glomeruli from C*fh*^−/−^ mice using laser dissection microscopy. Solublised glomerular isolates were then analysed using SDS-PAGE and C3 detected by western blotting using the polyclonal anti-mouse C3 antibody (Fig. 3b). As controls we loaded in the same gel plasma from wild-type and C*fi*^−/−^ mice to indicate the positions of the C3 *α*-chain (wild-type plasma) and the *α*-prime chain of C3b (CFI-deficient plasma). In C*fh*^−/−^ glomeruli isolates we could not detect evidence of either intact C3 *α*-chain or the *α*-prime chain of C3b (Fig. 3b). In contrast, the C3 *β*-chain was readily detectable together with *α*-chain fragments. This appearance was consistent with the presence of iC3b either alone or in combination with C3dg. In glomerular isolates from the C*fi*^−/−^ mice the C3 *β*-chain was also readily detectable. However, no *α*-chain fragments were evident indicating that neither iC3b nor C3dg was present in these glomeruli. On these gels there was a faint band running at a similar molecular weight to the intact C3 *α*-chain. Glomeruli from C*fh*^−/−^ mice reconstituted with mCFH demonstrated identical C3 fragments as those seen in the animals treated with PBS. Based on the immunostain and western blot analyses we concluded that C3dg bound to the GBM in the C*fh*^−/−^ mice was unaffected by the administration of mCFH.

We have previously shown that GBM C3 staining does not develop in mice with combined deficiency of CFH and CFI (C*fh*^−/−^.C*fi*^−/−^) (Rose et al., 2008). In these animals uncontrolled AP activation occurs but the generated C3b cannot be cleaved further due to the absence of CFI. However, when a source of mouse CFI (serum from mice with combined deficiency of factor H and C3) is administered to these animals not only does plasma C3b cleavage occur but GBM C3 staining becomes evident at 24 h using the anti-C3 antibody (Rose et al., 2008). In view of the present data we next examined the reactivity of the GBM C3 seen in CFI-reconstituted C*fh*^−/−^.C*fi*^−/−^ mice with the anti-C3d antibody (Fig. 3c). This showed an identical pattern of staining that seen in unmanipulated C*fh*^−/−^ mice, i.e. the GBM C3 was recognised by both antibodies.

### 3.3 mCFH does not interact with C3 deposited along the GBM

**Fig. 4:**
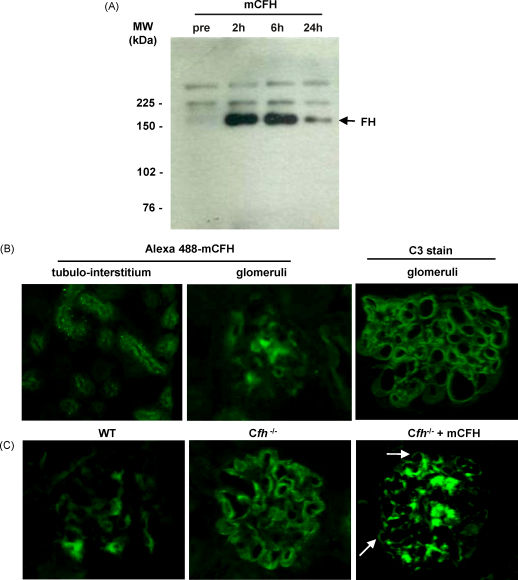
Detection of CFH-related proteins in C*fh*^−/−^ mice and tracking mCFH after administration to C*fh*^−/−^ mice. (A) Serum western blot for CFH using cross-reactive polyclonal anti-human CFH antibody. (B) Renal sections from C*fh*^−/−^ mice 2 h after the injection of Alexa-488-tagged CFH. At this time point the Alexa-488-tagged CFH was detected within the mesangium and tubulointerstitium but not along the GBM. At this time-point linear C3 staining is evident in C*fh*^−/−^ mice similar to unmanipulated C*fh*^−/−^ mice (far right panel). (C) Kidney sections immunostained for CFH using anti-human CFH antibody from wild-type, unmanipulated C*fh*^−/−^ mice and C*fh*^−/−^ mice 24 h after injection of mCFH. Some mesangial reactivity is present in wild-type glomeruli (left panel) whilst marked linear capillary wall staining is evident in the unmanipulated C*fh*^−/−^ mice (middle panel). In contrast, a mesangial staining pattern was evident in C*fh*^−/−^ mice 24 h after injection of mCFH (right panel) with very little staining along the capillary walls (arrows). Original magnification, 40×.

24 h after the injection of mCFH, we could still detect CFH in circulation by western blot but the maximal signal was 2 h following injection (Fig. 4a). To check whether the circulating mCFH could interact with glomerular C3 we tagged purified mCFH with Alexa 488 and assessed its distribution 2 h following the injection. We detected tagged CFH in the mesangium and within the tubulo-interstitium (Fig. 4b). This data showed that the administered mCFH did not directly interact with C3 bound on the GBM at this time point. Immunostaining of glomeruli from unmanipulated C*fh*^−/−^ mice showed reactivity with a polyclonal anti-CFH antibody in a linear pattern identical to that seen for C3 reactivity (Fig. 4c). We interpreted this reactivity as a consequence of cross-reactivity the anti-CFH antibody with CFH-related proteins that were associated with the GBM-bound C3 in the C*fh*^−/−^ mice. Notably, CFH-related protein staining was not detected along the GBM of wild-type mice. In glomerular sections from C*fh*^−/−^ mice reconstituted with mCFH a mesangial staining pattern was seen using the polyclonal anti-CFH antibody at 24 h. This pattern was identical to that seen for C3 using the anti-C3 antibody (Fig. 4c). These data suggested that the presence of CFH-related proteins on the GBM is associated with the presence of C3.

### 3.4 Administration of mCFH did not affect renal synthesis of C3

We considered that the appearance of C3 staining within the mesangium after mCFH administration could have been due to glomerular synthesis of C3. To test this hypothesis we performed real time-PCR assay to evaluate C3 mRNA expression in kidney tissue from C*fh* ^−/−^ mice injected with PBS or mCFH. No difference in C3 mRNA expression was detected between C*fh*^−/−^ mice injected with mCFH or PBS (data not shown) suggesting that the mesangial C3 staining was not a consequence of glomerular C3 synthesis.

### 3.5 Glomerular neutrophils in C*fh*^−/−^ mice that have received mCFH do not influence glomerular C3 changes and accumulate independently of CD11b (Mac-1)

**Fig. 5:**
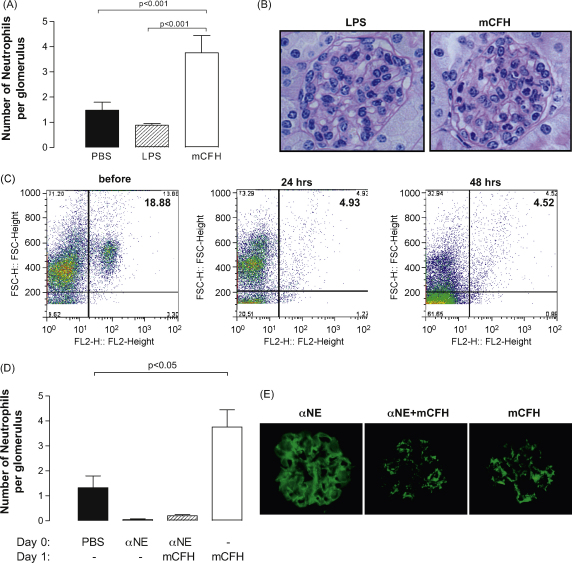
Influx of neutrophils into the glomeruli after the administration of mCFH. (A) Glomerular neutrophil numbers in C*fh*^−/−^ mice 24 h after injection of PBS, 0.75 μg of LPS or 1 mg of mCFH. Bars denote median values with standard deviation (B) Glomerular neutrophils evident in representative glomerular light microscopic images in mice that had received mCFH but not LPS. Original magnification 40× (C) FACS detection of blood neutrophils using rat IgG2b anti-mouse GR-1 antibody in *Cfh*^−/−^ mice before, 24 and 48 h after neutrophil depletion. Neutrophil depletion was achieved by a single injection of rat monoclonal IgG2b anti-murine neutrophil Ly.6G (αNE). (D) Glomerular neutrophil numbers in neutrophil-depleted C*fh*^−/−^ mice 24 h after injection of mCFH demonstrating absence of glomerular neutrophils in mice pre-treated with the αNE antibody. (E) Glomerular C3 stain using polyclonal anti-mouse C3 antibody in neutrophil-depleted mice treated with mCFH. The absence of neutrophils did not influence the change in glomerular C3 staining seen after the administration of mCFH. Compare this image with that shown in Fig. 2. Original magnification 40×. WT: wild-type.

Neutrophils were observed in the glomeruli of C*fh*^−/−^ mice 24 h after the injection of mCFH but not after injection of LPS alone (Fig. 5a and b). Human CFH has been reported to act as an adhesion ligand for neutrophils through CD11b (Mac-1) (DiScipio et al., 1998). To investigate whether the administration of mCFH could be directly involved in neutrophil recruitment we administered mCFH to C*fh*^−/−^ mice lacking CD11b (C*fh*^−/−^.CD11b^−/−^). 24 h after mCFH administration we observed significant glomerular neutrophil influx in these animals demonstrating that the neutrophil influx was independent of CD11b. To test if glomerular neutrophil proteases (Carlo et al., 1981), could influence glomerular C3 staining, we administered mCFH to C*fh*^−/−^ mice that had been depleted of neutrophils (Fig. 5c). The change in C3 staining pattern persisted despite neutrophil depletion indicating that neutrophils were not involved in C3 changes in the C*fh*^−/−^ mice following mCFH administration (Fig. 5d and e).

## 4 Discussion

Complement component C3 appears to be the culprit in DDD as deposition of C3 fragments derived from plasma is required for the renal lesion to develop (Pickering et al., 2002). So far specific therapy for controlling C3 activation remains unavailable. Plasma exchange therapy has been successfully used in a patient with DDD cause by C3 nephritic factor (Kurtz and Schlueter, 2002). Here we investigated the effect of mCFH in C*fh*^−/−^ mice which represent an experimental model of DDD (Pickering et al., 2002). Our results showed that the administration of mCFH was able to restore control of C3 activation in plasma, as evidenced by the appearance of intact C3 in the circulation of reconstituted C*fh*^−/−^ animals. Consistent with this observation was the reported increase in plasma C3 levels observed in a CFH-deficient individual following the administration of plasma (Nathanson et al., 2001). Furthermore, mCFH administration appeared to stop the deposition of C3 fragments along the GBM. Re-establishing control of alternative pathway activation, even if for a limited amount of time, resulted in an alteration in the pattern of C3 deposition within the kidney. In C*fh*^−/−^ mice C3 is normally detected along the GBM and absent within the mesangium and tubulo-interstitium. However, after administration of mCFH we detected C3 staining within both the mesangium and tubulo-interstitium of C*fh*^−/−^ mice together with alteration in C3 staining along the GBM.

Tubulo-interstitial staining for C3 is present in healthy wild-type mice. It appears to require the ability to activate the alternative pathway as it is absent in factor B-deficient mice (Lenderink et al., 2007). In unmanipulated C*fh*^−/−^ mice it is absent while restoring some degree of plasma C3 regulation in C*fh*^−/−^ mice through the administration of mCFH we consistently detected C3 staining within the tubulo-interstitium. This data is consistent with renal transplant studies where C*fh*^−/−^ kidneys have been placed into wild-type hosts (Alexander et al., 2007). In these experiments complete resolution of GBM C3 staining was seen with concomitant appearance of normal tubulo-interstitial C3 staining pattern. When the opposite experiment was performed, i.e. wild-type kidneys placed into C*fh*^−/−^ hosts, tubulo-interstitial C3 staining within the wild-type transplanted kidney was lost. Thus, this data together with our results suggest that the normal C3 staining within the tubulo-interstitium is dependent on the presence of intact circulating C3.

The presence of mesangial C3 was evident in C*fh*^−/−^ mice reconstituted with mCFH using a polyclonal anti-C3 antibody. However, this staining was not detectable using a polyclonal anti-C3d antibody. Previous experimental animal data has suggested that, in situations where there is dysregulation of the alternative pathway activation, the nature of the C3 activation fragment produced in plasma is important in determining where it deposits within the glomerulus. In C*fi*^−/−^ mice excessive production of activated C3b occurs and the absence of CFI prevents further degradation of C3b to its metabolites: iC3b, C3c and C3dg (Rose et al., 2008). In these animals abnormal mesangial C3 staining is evident using the same polyclonal anti-C3 antibody used in this study (Rose et al., 2008). However, this mesangial C3 does not react with the polyclonal anti-C3d antibody (Leung et al., unpublished data). Taken together with our laser capture analysis of C3 in glomeruli from C*fi*^−/−^ mice, this data indicated that the anti-C3d antibody, in contrast to the anti-C3 antibody, could not recognise C3b in renal sections. Furthermore, when the anti-C3d antibody was used in a sandwich ELISA it failed to detect intact plasma C3 in wild-type mice (Leung et al., unpublished data). Based on lack of reactivity with the anti-C3d antibody we postulated that the mesangial C3 reactivity observed in C*fh*^−/−^ mice reconstituted with mCFH could represent (1) intact C3 derived from local synthesis, (2) C3b derived from the circulation (analogous to that seen in C*fi*^−/−^ mice) or (3) C3c which lacking the C3dg fragment would be expected not to react with anti-C3d antibody. We considered the first possibility unlikely since we could not detect any evidence of an increase in renal C3 synthesis following administration of mCFH by RT-PCR. However, it could be that our quantitative C3 RT-PCR analysis using kidney tissue may not have been sensitive enough to detect a minor increase in mesangial C3 synthesis. We excluded the second possibility since we could not detect evidence of C3b in either the plasma or laser dissected glomeruli of C*fh*^−/−^ mice reconstituted with mCFH. It therefore seemed most likely that the mesangial C3 in the reconstituted animals represented the C3c fragment. This is consistent with data from human DDD studies that have shown that paramesangial C3 deposits appear to be comprised of C3c only (West et al., 2001).

Our data also showed differences in the GBM reactivity using the two anti-C3 antibodies in the C*fh*^−/−^ mice reconstituted with mCFH. In unmanuipulated C*fh*^−/−^ mice linear capillary wall staining for C3 was evident using both the anti-C3 and anti-C3d antibody (Fig. 3a). Analysis of glomerular C3 using laser capture microscopy demonstrated the presence of the C3 *β*-chain together with *α*-chain fragments (Fig. 3b), an appearance consistent with iC3b. This demonstrated that iC3b is present within the GBM of C*fh*^−/−^ mice. The reactivity also suggested that the anti-C3d antibody could recognise iC3b in addition to C3dg. However, we could not exclude that C3dg in addition to iC3b was present along the GBM since the molecular weight of C3dg (38 kDa) is similar to the molecular weight of the *α*-chain fragments of iC3b (43 and 40 kDa). Hence the presence of both iC3b and C3dg along the GBM would have given the same bands on the glomerular western blot analysis as iC3b alone. Following administration of mCFH there was a striking reduction in GBM reactivity using the anti-C3 antibody whereas the intensity of GBM staining using the anti-C3dg antibody remained unchanged. This differential reactivity indicated that the nature of the C3 along the GBM had changed. The lack of change in the intensity of the anti-C3d reactivity could indicate that mCFH administration did not alter the degree of GBM associated C3d. However, this would not explain the concomitant appearance of C3c within the mesangium following mCFH administration. An alternative possiblity was that following temporary restoration of plasma C3 regulation in the C*fh*^−/−^ animals reconstituted with mCFH iC3b bound along the GBM was cleaved into C3dg and C3c fragments, the former remaining bound to the GBM and the latter removed to mesangial areas. Initially we considered this processing could be mediated by exogenous mCFH together with CFI. However, our data showed that the injected mCFH did not co-localize with iC3b along the GBM. Furthermore, CFH is not considered to be able to act as a cofactor for the CFI-mediated cleavage of iC3b under physiological conditions (Sim et al., 1993). Therefore, it appeared that if further processing of GBM-bound C3 occurred it was not due to a direct effect of the injected mCFH. Administration of CFH also induced migration of neutrophils in to the glomeruli. We considered that these could be involved in the cleavage of iC3b along the GBM. Bound iC3b is highly sensitive to proteases and neutrophil elastase are extremely active in cleaving iC3b to C3c and C3dg (Carlo et al., 1981). However, the appearance of mesangial C3 reactivity persisted despite neutrophil depletion. The explanation for the glomerular neutrophil influx seen in mCFH-reconstituted animals is not clear. CFH could theoretically facilitate the migration of neutrophils via interaction with the integrin CD11b independently of iC3b (Avery and Gordon, 1993; DiScipio et al., 1998). However, glomerular neutrophils still appeared following mCFH adminstration in C*fh*^−/−^ animals that lacked CD11b. We were unable to test whether neutrophil influx developed using mCFH that was completely free of LPS so it remains possible that this phenomenon was a synergistic effect of mCFH and LPS.

We also considered the possibility that CFH-related proteins could be involved in the processing of GBM-bound C3. In unmanipulated C*fh*^−/−^ mice we detected capillary wall staining pattern using a polyclonal anti-CFH antibody. As these animals lack CFH this reactivity represented the presence of CFH-related proteins (CFHRs) along the GBM, co-localizing with C3. It is noteworthy here that the presence of the CFHR protein, CFHR-5, has been detected in 92 out of 100 biopsies from patients with glomerular sclerosis from all causes, including diabetic nephropathy, focal glomerular sclerosis and advanced proliferative glomerular diseases with areas of sclerosis (Murphy et al., 2002). In all of them CFHR-5 had a similar pattern of distribution to C3. Furthermore, CFHR-5 was strongly associated with complement-containing glomerular immune deposits (Murphy et al., 2002).

In summary, the administration of mCFH primarily restored plasma C3 regulation and this subsequently favored the clearance of the GBM-bound iC3b. Our data suggested that, whilst iC3b within the GBM was removed, C3dg remained bound to the GBM. We were unable to detect localisation of the exogenous mCFH with GBM C3 indicating that the alteration in GBM C3 was not due to a direct effect of mCFH. This processing of GBM C3 could be mediated by CFH-related proteins. We propose that that CFH acts primarily to down-regulate C3 activation in plasma. Within the GBM the CFH-related proteins are important in the intra-renal physiological processing of any deposited iC3b. However, their function is overridden when excessive iC3b is produced in circulation that accumulates along the GBM. Our data reinforce the concept that DDD therapy should be directed at interventions that restore plasma C3 regulation.
